# Traumatic brain injury recovery prediction by harmonizing real brain CT and synthetic brain MRI: a pilot study

**DOI:** 10.1093/braincomms/fcag123

**Published:** 2026-04-06

**Authors:** Yiming Che, Amogh Manoj Joshi, Jay Shah, Md Mahfuzur Rahman Siddiquee, Catherine D Chong, Simona Nikolova, Gina Dumkrieger, Baoxin Li, Teresa Wu, Todd J Schwedt

**Affiliations:** School of Computing and Augmented Intelligence, Arizona State University, Tempe, AZ 85281, USA; ASU-Mayo Center for Innovative Imaging, Tempe, AZ 85281, USA; School of Computing and Augmented Intelligence, Arizona State University, Tempe, AZ 85281, USA; ASU-Mayo Center for Innovative Imaging, Tempe, AZ 85281, USA; School of Computing and Augmented Intelligence, Arizona State University, Tempe, AZ 85281, USA; ASU-Mayo Center for Innovative Imaging, Tempe, AZ 85281, USA; School of Computing and Augmented Intelligence, Arizona State University, Tempe, AZ 85281, USA; ASU-Mayo Center for Innovative Imaging, Tempe, AZ 85281, USA; ASU-Mayo Center for Innovative Imaging, Tempe, AZ 85281, USA; Department of Neurology, Mayo Clinic, Phoenix, AZ 85054, USA; Department of Neurology, Mayo Clinic, Phoenix, AZ 85054, USA; Department of Neurology, Mayo Clinic, Phoenix, AZ 85054, USA; School of Computing and Augmented Intelligence, Arizona State University, Tempe, AZ 85281, USA; ASU-Mayo Center for Innovative Imaging, Tempe, AZ 85281, USA; School of Computing and Augmented Intelligence, Arizona State University, Tempe, AZ 85281, USA; ASU-Mayo Center for Innovative Imaging, Tempe, AZ 85281, USA; ASU-Mayo Center for Innovative Imaging, Tempe, AZ 85281, USA; Department of Neurology, Mayo Clinic, Phoenix, AZ 85054, USA

**Keywords:** deep learning, traumatic brain injury, concussion, neuroimaging, data harmonization

## Abstract

To enhance the prediction of traumatic brain injury (mTBI) outcomes, we propose a deep learning approach that integrates brain computed tomography (CT) scans with corresponding synthetic T1-weighted magnetic resonance imaging (T1-MRI). Our method significantly outperforms the prediction using CT scans alone. TRACK-TBI Pilot dataset, which includes imaging and clinical outcome data from patients with TBI, is studied. The hypothesis is brain CT and T1-MRI complement each other and together will improve TBI prognosis compared to using either CT or T1-MRI alone. Since CT and T1-MRI may not be available for the same individual, we employed a specialized version of a generative adversarial network (GAN), known as fixed-point GAN (FP-GAN). FP-GAN was trained using unpaired CT and T1-MRI scans to generate synthetic T1-MRIs from real CT scans. This process produced pseudo-paired CT-MRI data, which was then used to train a deep learning classifier for outcome prediction. The classifier consists of dual parallel 3D ResNet-18 models, each independently processing T1-MRI and CT scans. We used Glasgow Outcome Scale-Extended (GOSE) scores at 3 months post-TBI as the measure of patient outcomes. To avoid data leakage, the subjects used in FP-GAN and ResNet-18 model have no overlap. We further divided the paired data, allocating 69 samples for 5-fold cross-validation and 17 samples for testing. Prognostic performance was evaluated using the area under the receiver operating characteristic curve (AUC), *F*1-score (the harmonic mean of precision and recall), sensitivity (true positive rate) and specificity (true negative rate). For binary classification, we defined good recovery as GOSE ≥ 7 (positive) and poor recovery (negative) as 3 ≤ GOSE ≤ 6. Accordingly, our training set consists of 24 subjects with poor recovery and 45 subjects with good recovery, while the testing set includes 5 subjects with poor recovery and 12 subjects with good recovery. A DeLong test on AUC confirms that the improvement from incorporating synthetic T1-MRI (AUC = 0.76 ± 0.10) is statistically significant (*P* < 0.05) compared to using CT alone (AUC = 0.68 ± 0.13). The significant improvement from using the combination of real CT and synthetic T1-MRI in sensitivity (SEN = 0.95 ± 0.07) and overall performance metrics, such as *F*1-score (*F*1 = 0.84 ± 0.03), suggests that the proposed approach provides a robust and effective prognostic approach compared to using CT alone (SEN = 0.83 ± 0.18 and *F*1 = 0.76 ± 0.07). This pilot research demonstrates the potential of a deep learning-based harmonization model to bridge the gap between CT and T1-MRI in TBI assessment. By integrating synthetic T1-MRI with CT, prediction performance is substantially enhanced.

## Teaser

Che *et al.* demonstrated that combining brain computed tomography with synthetic magnetic resonance imaging generated by a deep learning model significantly improved the prediction of traumatic brain injury outcomes, compared to computed tomography alone, highlighting the potential of cross-modality image synthesis to enhance prognostic accuracy when magnetic resonance imaging is unavailable.

## Graphical Abstract

**Graphical Abstract:**
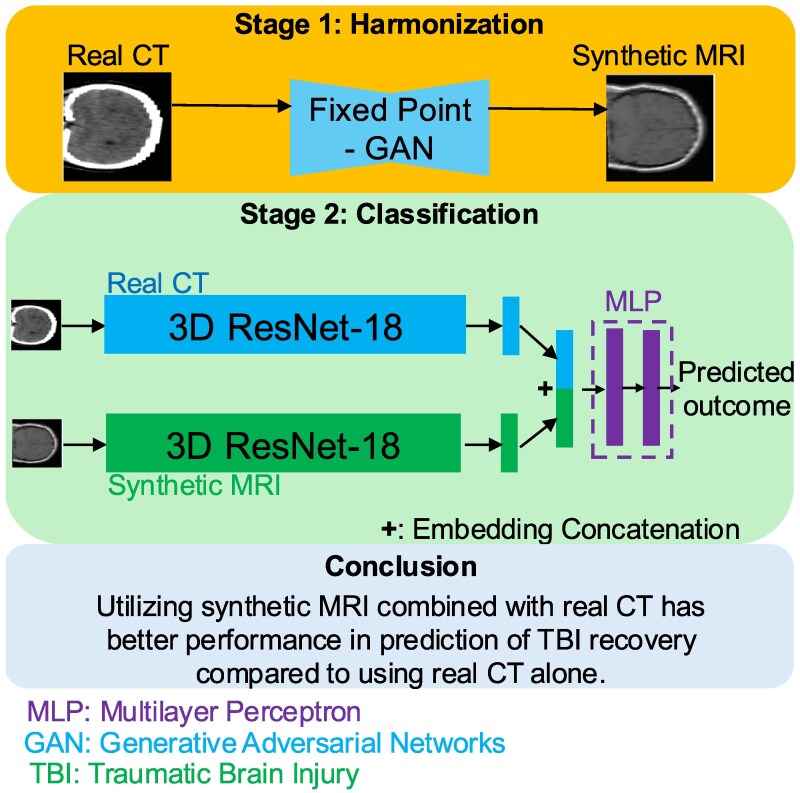

## Introduction

Traumatic brain injury (TBI) is among the leading causes of death and disability in the USA and globally.^1^ It is estimated that over 55 million individuals experience a ‘mild’ TBI (mTBI) annually, while 5.5 million endure ‘severe’ TBI.^2^ The lifetime economic burden, including healthcare costs, lost productivity and diminished quality of life, is substantial. For the ∼2 million people who suffer from TBI each year in the USA, the financial impact is estimated to reach $750 billion.^3^ Early and accurate prognostication of short- and long-term patient outcomes following TBI would help determine the most appropriate intensity and frequency of clinical management. For example, many patients with mTBI have complete resolution of symptoms within hours or days of their injury and thus require minimal intervention and follow-up. However, other patients who have very similar initial manifestations of their mTBI have persistent and disabling symptoms that can last for months or even years. Although evidence-based treatment options for mTBI are currently limited, early post-mTBI treatment and close follow-up might lessen the post-mTBI burden for patients likely to have persistent symptoms.

The Glasgow Outcome Scale-Extended (GOSE) is commonly used to assess patient outcomes following TBI,^4^ and it is a recommended outcome measure by the National Institute of Neurological Disorders and Stroke. Notably, it is the only primary outcome measure accepted by the U.S. Food and Drug Administration for use in TBI research supporting New Drug Applications.^5^ Multiple studies have developed models to predict future GOSE scores, utilizing varying input features such as age, Glasgow Coma Scale motor score, Marshall CT score, pupillary dilatation, posttraumatic confusional state duration and CT or magnetic resonance imaging (MRI) findings^6-8^. These studies have demonstrated the potential for brain imaging findings, collected early after a TBI, to meaningfully contribute to the prediction of future GOSE scores. CT scans have already been investigated for outcome prediction in patients with severe TBI.^9^ CT is the preferred imaging modality in acute head trauma, as it accurately detects and facilitates the treatment of extra- and intracranial haemorrhage, hydrocephalus, mass effect and vascular injuries.^10^ When significant symptoms persist, MRI becomes the modality of choice in the subacute and chronic phases of TBI, providing greater sensitivity to subtle or delayed brain injuries that may not be visible on CT.^10^ Hence, CT and MRI complement each other^10^ and a prediction of patient outcomes that solely depends on CT scans may be suboptimal.

We hypothesized that a multimodal approach utilizing both CT and MRI may improve TBI outcome prediction. For example, a recent multicentre study of mTBI found that abnormal head CT findings alone were not significantly predictive of 3-month outcomes, whereas the presence of lesions on early MRI such as small haemorrhagic contusions or multiple axonal injuries was strongly associated with worse recovery.^11^ Incorporating MRI findings alongside clinical and CT data substantially improved the accuracy of prognostic models in that study. This evidence supports the intuition that if both CT and MRI could be available for a given patient, outcome prediction might be more accurate than using either modality in isolation. However, in the clinical setting, both CT and MRI are rarely available for the same patient. This limitation has spurred interest in synthetic or ‘harmonized’ imaging approaches, wherein one modality is computationally generated from the other to create a unified multimodal dataset. Recent advances in deep learning, particularly generative adversarial networks (GANs), have made it possible to translate images from one domain to another (e.g. CT to MRI) while preserving anatomical content. GAN-based methods have been applied in various neuroimaging contexts to augment or substitute imaging modalities—for instance, generating MRI scans from CT to aid radiotherapy planning and image registration,^12^ or producing synthetic MRI sequences when certain MRI contrasts are missing.^13^ Notably, GAN-generated synthetic images have demonstrated utility comparable to real images in some tasks, suggesting that they can enhance downstream analyses or predictions.^14^

Motivated by these findings and to bridge the gap between CT and MRI availability in clinical practice, we implemented FP-GAN as our harmonization model to render synthetic MRIs from CTs and developed a multimodal prognostic model using CT and synthetic MR for TBI outcome prognosis. In our study, harmonization refers specifically to the deep learning–based image-to-image translation from CT to MRI, with the goal of generating synthetic MRI scans from CT inputs. This differs from the conventional use of harmonization in MRI research, such as Combat,^15-19^ which typically addresses scanner- or site-related variability within the same modality and focuses on harmonizing derived imaging features rather than the whole-brain image. We contend our approach involves cross-modality synthesis to enrich the dataset and extends the concept of harmonization beyond traditional boundaries and offers a novel pathway to integrate multimodal neuroimaging for improved TBI outcome prediction.

## Materials and methods

### FITBIR and TRACK-TBI Pilot

In 2012, the U.S. Department of Defense (DoD) and the National Institutes of Health (NIH) launched a collaborative effort to establish tFITBIR (Federal Interagency Traumatic Brain Injury Research) database. FITBIR contains a wealth of data, including clinical measures, outcomes and neuroimages. Our comprehensive evaluation of FITBIR data identified three multi-site projects that had CT and/or MRI: TRACK-TBI Pilot, TRACK-TBI Adult and CARE. Most research has focused on analyzing the imaging data from each study in isolation, missing the opportunity to examine the reliability and reproducibility of findings across different studies and the opportunity to create a larger pooled dataset that maximizes statistical power. Pooling multimodal neuroimaging from different studies is challenging because (i) for each modality, CT or MRI, there exist variations in scanners, scanning protocols and image post-processing techniques; and (ii) not all the studies have both CT and MRI modalities available. In this research, we conduct a pilot study on the application of imaging translation techniques to harmonize CT and T1-weighted MRI (T1-MRI) and demonstrate the advantages of a multimodal model in TBI study. We focus on the TRACK-TBI Pilot since it provides both CT and T1-MRI from the same research participant. We use the abbreviation MRI instead of T1-MRI for simplicity in the following sections.

### GAN as harmonization technique for synthetic imaging rendering

**Figure 1:**
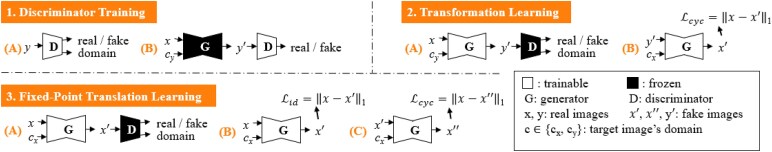
**FP-GAN training scheme.** ^26^ The discriminator learns to distinguish real or synthesized images and classify the domains of input images (1A–1B). The generator learns to perform not only cross-domain translations via transformation learning (2A–2B), but also same-domain translations via fixed-point translation learning (3A–3C).

GAN-based models are being utilized for medical imaging applications, particularly for translating between different imaging modalities. For example, standard GAN models facilitate MRI to CT translations,^20^ conditional GAN (cGAN)^21^ models are employed for translating CT scans into PET scans^22^ and MRI,^23^ Cycle-GANs^24^ are commonly used for MRI-to-CT translation and vice versa.^25^ We adopted the fixed-point generative adversarial network (FP-GAN), a bidirectional unpaired data translation for synthetic imaging rendering. The FP-GAN is an advanced iteration of the Cycle-GAN, incorporating revised adversarial domain classification that significantly boosts performance, enhancing the accuracy and reliability of image translations compared to other GAN-based models.^26^ As shown in Fig. 1, the architecture of the FP-GAN consists of two primary components: the generator and the discriminator, both tailored for tasks involving conditional image generation. The generator processes the input image and domain information through a series of convolutional, residual and upsampling layers, ultimately synthesizing an output image, while preserving spatial features through six residual blocks. The discriminator consists of six convolutional layers that downsample the image, with dual outputs for source identification (real or fake) and domain classification, facilitating both accurate authenticity and class assessment.

A significant improvement is the introduction of same-domain translation training in FP-GAN, which regularizes cross-domain translation by encouraging the generator to identify a minimal transformation function while penalizing any changes that do not contribute to the domain translation task. This innovative approach delivers three key advantages: enhanced same-domain translation, regularized cross-domain translation and source-domain-independent translation. The comprehensive loss functions ensure that the adversarial loss encourages the generation of realistic images, the domain classification loss confirms images are from the correct domain in both same- and cross-domain scenarios and the cycle consistency loss guarantees that any generated image can be accurately translated back to its original domain. FP-GAN incorporates fixed-point translation learning, ensuring that same-domain images remain unchanged and that cross-domain transformations are limited to only necessary modifications. This is particularly crucial in medical imaging, where anatomical accuracy must be maintained. Moreover, by enforcing conditional identity loss, FP-GAN enhances translation reliability, leading to more precise harmonization. These advantages make FP-GAN a more suitable choice for harmonizing MRI and CT data while preserving clinically relevant details.

It is important to recognize that in clinical settings, access to both imaging modalities may not always be possible, presenting challenges for comprehensive assessment and model application. To address the challenge of simultaneously accessing both MRI and CT imaging modalities, we have integrated FP-GAN for unpaired data harmonization. We used the hyperparameters: batch size 16, learning rate $10^{-4}$ and 30 000 training iterations. The generator and discriminator used here have the same structures as the original FP-GAN. The weights of the loss function terms remain unchanged from the original FP-GAN, except for the identity loss term. We set the identity loss weight to 0.1, in contrast to the original value of 10, to encourage more substantial domain translation. The model is trained on Nvidia-A100 GPU @ 80 GB. Although FP-GAN is inherently designed for bidirectional translation, this study focuses solely on CT-to-MRI translation since it is much more common for brain CT to be completed during the clinical evaluation of mTBI.

### Deep learning prognostic model

**Figure 2:**
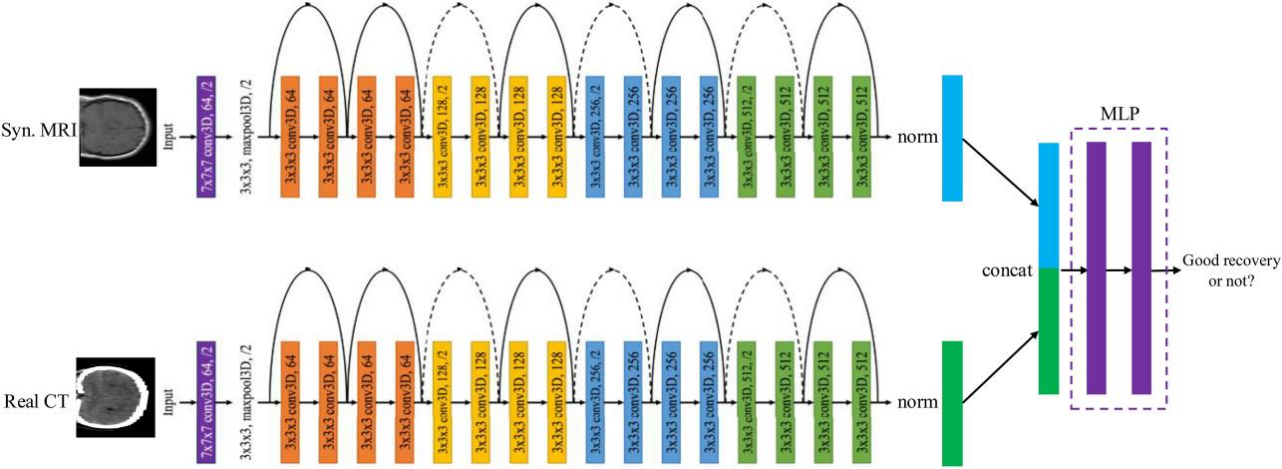
**The structure of our prognostic model.** It employs two 3D ResNet-18s and concatenates features from both modalities at the end of ResNet-18 after normalization (norm) for further classification using a two-layer MLP.

A prognostic model that employs dual parallel 3D ResNet-18 (shown in Fig. 2) is designed to independently process MRI and CT scans. ResNet^27^ models, noted for their robust performance, are extensively utilized for tasks such as early diagnosis of Alzheimer’s disease,^28^ differentiation of benign versus malignant tumors,^29^ subtype classification of haemorrhages^30^ and skin lesion detection.^31^ These studies underscore the capability of ResNet models in accurately identifying a variety of diseases through medical imaging. Compared to traditional statistical models used in medical applications,^32,33^ DL models can better handle complex data structures, e.g. image, due to their greater model capacity.

The integration of the features from both 3D ResNet-18 models is accomplished through concatenation, allowing for a comprehensive feature fusion that leverages the distinct characteristics of each imaging modality. To be more specific, the two 512-dimensional embeddings, illustrated by the green and blue blocks in Fig. 2, are extracted from separate 3D ResNet-18 models, which encode the CT and MRI volumes, respectively. Each ResNet-18 outputs a 512-dimensional feature vector after the global average pooling layer. These embeddings are first L2-normalized, scaling each vector to unit norm to preserve directional information while removing the effect of magnitude. This normalization facilitates more stable and meaningful computations. The normalized embeddings are then concatenated to form a 1024-dimensional combined representation (purple block in Fig. 2), which is subsequently passed through a multi-layer perceptron (MLP) for outcome prediction.

We also tested alternative modality fusion methods, which are presented in the Discussion section. The prognostic model uses outcomes derived from GOSE as response. GOSE categorizes patient outcomes into eight levels: 1—death, 2—vegetative state, 3—lower severe disability, 4—upper severe disability, 5—lower moderate disability, 6—upper moderate disability, 7—lower good recovery and 8—upper good recovery. In this pilot study, we decided to focus on patients with GOSE scores >2 because the prediction of these outcomes is more clinically challenging. We categorize good recovery (positive) as GOSE scores of 7–8, contrasting with scores of 3–6 (negative) for a binary classification.

### TRACK-TBI Pilot datasets

Our Data Access Request to FITBIR database was approved on 28 February 2023. TRACK-TBI Pilot dataset was downloaded from FITBIR. The TRACK-TBI Pilot, a prospective, multi-centre study, recruited patients experiencing head trauma from external forces. Participants were admitted to one of three participating U.S. Level 1 trauma centres and underwent a head CT within 24 h of injury, following protocols as described in the Clinical-Trials.gov (Registration: NCT01565551).

**Figure 3:**
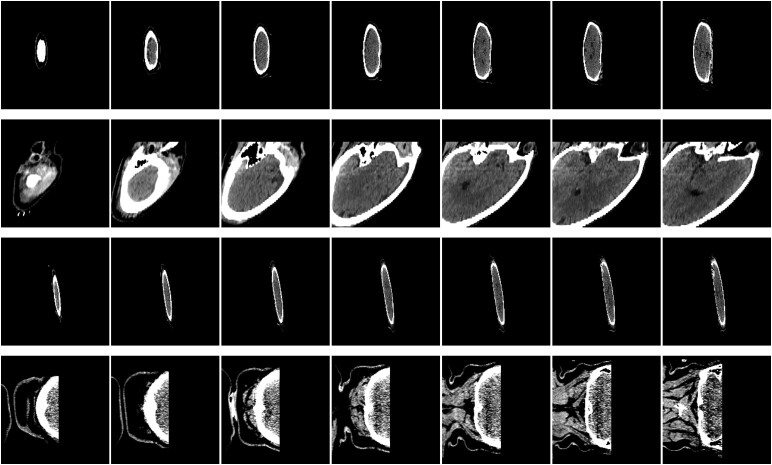
**The four removed samples due to quality issue.** The four rows correspond to four samples, while each column displays axial slices from the respective samples.

All images were converted to the Neuroimaging Informatics Technology Initiative (NIFTI) format. In our preprocessing pipeline, we first registered the images with the MNI152 template with 1 mm resolution using the linear registration tool FLIRT.^34^ The voxel intensities of all the NIFTI images rescaled to [0,1] which is a standard preprocessing step in deep learning to ensure numerical stability and consistency across inputs and then converted into two-dimensional (2D) images by slicing across axial view for data harmonization. The TRACK-TBI dataset includes 599 subjects, of whom 428 have GOSE information at 3 months post-mTBI. Among them, 222 subjects have only CT scans (Dataset 1: DS1), 37 have only MRI scans (Dataset 2: DS2) and 83 have paired CT and MRI with GOSE information at 3 months (Dataset 3: DS3 mixed) and 74 of DS3 mixed are mTBI patients (Dataset 3: DS3 mTBI) (see Table 1). Here, Glasgow Coma Scale^35^ (GCS) score at emergency department (ED) is also included in Table 1 for reference. The remaining 11 patients in DS3 mixed have severe TBI. Finally, 86 samples are dropped due to the quality issue. The following types of artefacts led to exclusion: (i) motion artefacts causing duplicated or ghosted structures across slices, (ii) partial brain coverage or missing slices and (3) excessive noise obscuring tissue contrast. We have added four representative examples of excluded samples in Fig. 3, which illustrates the degree of artefact severity that led to exclusion.

**Table 1:** Summary of TRACK-TBI Pilot data used in this study

Dataset: Modality	No. of patients	Age	Sex	GOSE	GCS (ED)
DS1: Unpaired CT	222	48.4±19.7	M:152, F:70	6.0±2.0	13.7±3.0
DS2: Unpaired T1-MRI	37	39.6±16.0	M:33, F:4	6.5±1.6	13.8±2.9
DS3: Paired CT and T1-MRI (mixed)	83	39.6±16.2	M:52, F:31	6.8±0.9	14.0±2.4
DS3: Paired CT and T1-MRI (mTBI)	74	40.1±16.5	M:43, F:31	7.0±1.0	14.8±0.3

Unlike the classification model, which operates on 3D volumes, FP-GAN was trained using 2D slices extracted from the 3D CT and MRI data. We employed a set of slice-level quantitative criteria to identify images with insufficient anatomical content or clear acquisition artefacts. For both CT and MRI, this included basic intensity statistics and spatial features such as signal variation, entropy (as a proxy for information content), edge-based sharpness (to detect blurred or low-detail images) and the proportion of foreground pixels relative to background. For MRI, we further analysed the spatial organization of signal by verifying that a meaningful connected anatomical region occupies a reasonable area of the image and that relevant signal is present near the centre of the field of view, enabling detection of partial scans, off-centre acquisitions and slices dominated by background or peripheral artefacts. For CT, a conservative strategy was adopted, whereby only slices that were effectively empty (i.e. dominated by background with negligible foreground signal) were excluded. In total, 1410 out of 12 325 CT slices (11.4%) and 681 out of 8067 MRI slices (8.4%) were removed.

We used DS1 and DS2 to train FP-GAN for generating synthetic MRIs from CT scans. For prognosis tasks, we employed two subsets from DS3 (mTBI), which includes only mild TBI patients and DS3 (mixed), which includes patients with mild, moderate and severe TBI. In DS3 (mixed), 66 samples (23 with poor recovery and 43 with good recovery) were used for 5-fold cross-validation (CV), and an additional 17 samples (5 poor recovery, 12 good recovery) were reserved for blind testing. For DS3 (mTBI), 60 samples (18 poor recovery, 42 good recovery) were used in 5-fold CV, with 14 samples (4 poor recovery, 10 good recovery) held out for blind testing.

We conducted TBI recovery prognosis under five distinct settings for both DS3 mixed and DS3 mTBI datasets: using (i) real CT data from 86 patients with corresponding synthesized MRI data for testing our dual parallel ResNet; (ii) real CT; (iii) synthesized MRI; (iv) real MRI data and (v) real CT and MRI data. Note that experiments (1)–(5) share the same 86 mixed participants for DS3 mixed and 75 mTBI participants for DS3 mTBI. Following data harmonization, prediction was performed on the harmonized 3D volumetric data for each patient, which involved concatenating synthesized 2D data into a 3D volume. To ensure the robustness and generalizability of our model, we implemented a 5-fold CV strategy across all settings to verify the prognosis outcomes. Finally, we report the averaged results from 5-fold CV. We resampled samples with bad recovery to match the number of samples with good recovery during training since the dataset is imbalanced.

### Statistical analysis

Comparisons between receiver operating characteristic curves (AUCs) were conducted using the DeLong test^36^ for correlated ROC curves. A two-sided *P*-value < 0.05 was considered statistically significant.

## Results

### FP-GAN for synthetic MRI generation

**Figure 4:**
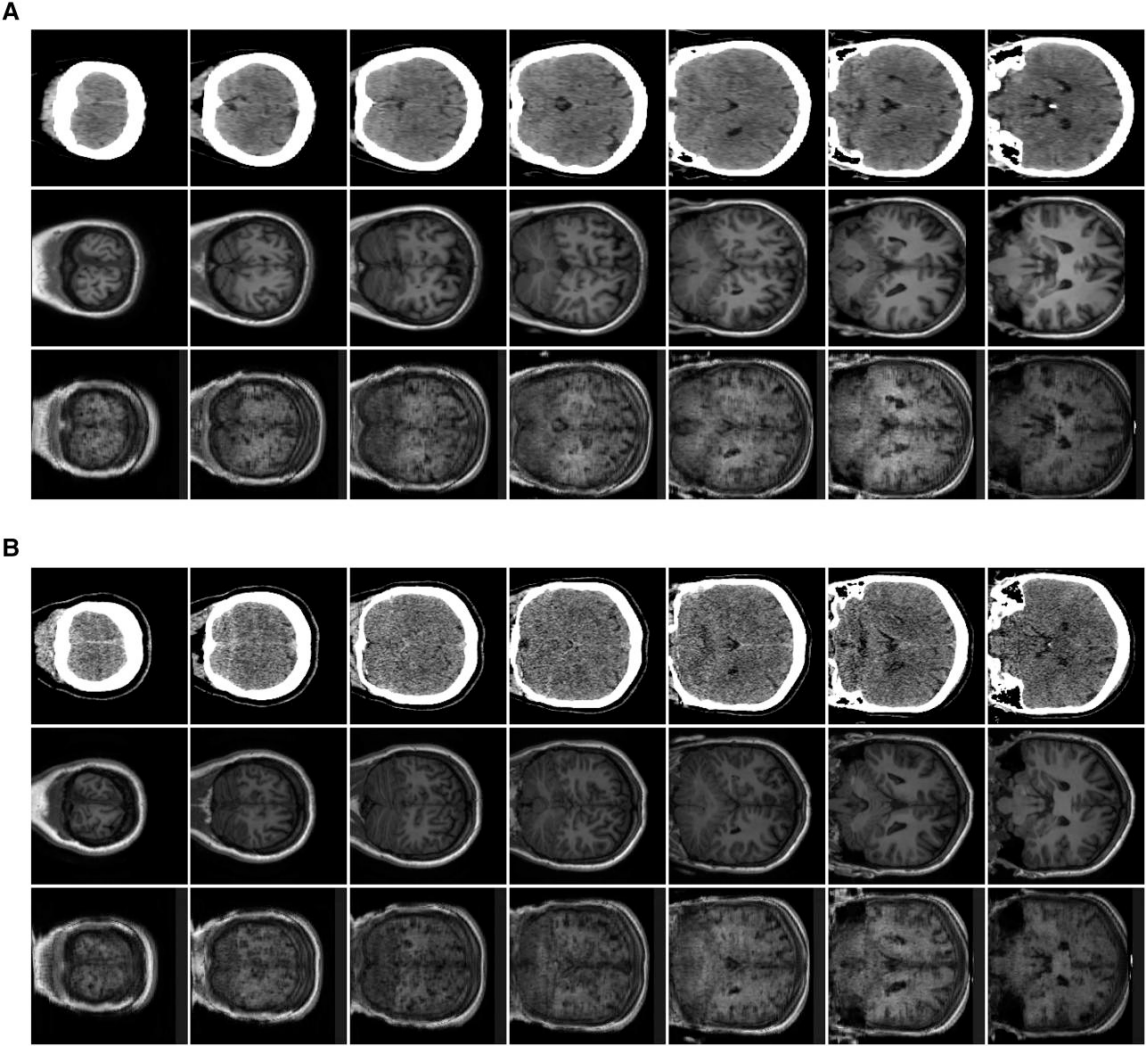
**Qualitative results of imaging translation of two subjects (labelled as A and B) using FP-GAN.** For each subject, the three rows correspond to real CT, real MRI and synthetic MRI, while each column displays axial slices from the respective modality.

The qualitative visual results of the harmonization (Fig. 4) showcase two representative examples. The synthetic MRIs demonstrate well-preserved anatomical details, indicating the effectiveness of our harmonization approach.

The FP-GAN harmonization model was trained using unpaired CT and MRI data, specifically, 222 CTs (DS1) and 37 MRIs (DS2), which do not come from the same individuals. This setting reflects a real-world scenario where CT and MRI are often not available for the same patient and follows the standard FP-GAN framework for unpaired image-to-image translation. For quantitative evaluation of the harmonization model’s performance, we used a separate set of paired CT and MRI scans from 86 participants (DS3 mixed). These paired images were not used during training, ensuring an unbiased assessment.

We computed the average L1 distance and Structural Similarity Index Measure (SSIM) between synthetic and real MRI images. The average L1 distance was 0.182, indicating pixel-level differences between the two modalities, while the SSIM was 0.365, reflecting moderate structural similarity. L1 distance captures absolute intensity differences, whereas SSIM evaluates perceptual similarity based on luminance, contrast and structure. These metrics provide complementary insights into the quality of synthetic MRI. We further elaborate on the synthetic image generation process and its limitations in the Discussion section.

### Prediction of mTBI outcomes

The prediction results from DS 3 mixed (Table 2) indicate that combining synthetic MRI with CT (Exp IV) significantly outperforms CT alone (Exp I) across all metrics (0.76 versus 0.68 in AUC, 0.84 versus 0.76 in *F*1, 0.95 versus 0.83 in Sensitivity and 0.24 versus 0.20 in Specificity). A DeLong test on AUC confirms that the improvement from incorporating synthetic MRI is statistically significant (*P* < 0.05). In addition, the metrics have smaller standard deviations in the real CT combined with synthetic MRI setting versus real CT alone indicating the improved robustness of the model. Additional experiments using only synthesized MRI (Exp III) or real MRI (Exp II) demonstrate that combining real CT yields a notable performance boost over using either modality alone. The synthesized MRI is generated from the corresponding CT, and the harmonization model is trained in an unpaired manner. Consequently, the synthesized MRI can only capture a coarse representation of the patterns from the training MRI data, making it complementary to the corresponding CT.

**Table 2:** Prognostic results of the five distinct settings from DS3 (mixed)

Experiments	AUC	*F*1	Sensitivity	Specificity
Exp I: Real CT	0.68±0.12	0.75±0.06	0.82±0.19	0.22±0.29
Exp II: Real MRI	0.69±0.11	0.73±0.07	0.74±0.18	0.37±0.32
Exp III: Synthetic MRI	0.58±0.06	0.63±0.11	0.59±0.21	0.46±0.26
Exp IV: Real CT + Synthetic MRI	0.77±0.08	0.83±0.04	0.94±0.07	0.26±0.21
Exp V: Real CT + Real MRI	0.73±0.10	0.84±0.02	0.97±0.04	0.15±0.14

We further refined the dataset by excluding non-mTBI patients to create DS3 (mTBI) and conducted the same classification task as with DS3 (mixed) (results in Table 3). We observed that all experiments have similar discriminative power, i.e. AUC. However, the experiments with either synthetic or real MRI obtain much higher *F*1 and sensitivity compared to the experiment with CT only. The classification results on DS3 (mixed) and DS3 (mTBI) are further discussed in the Discussion section.

**Table 3:** Prognostic results of the five distinct settings from DS3 (mTBI)

Experiments	AUC	*F*1	Sensitivity	Specificity
Exp I: Real CT	0.68±0.19	0.55±0.28	0.53±0.28	0.49±0.27
Exp II: Real MRI	0.69±0.09	0.84±0.04	0.97±0.04	0.24±0.17
Exp III: Synthetic MRI	0.65±0.08	0.76±0.12	0.81±0.21	0.27±0.29
Exp IV: Real CT + Synthetic MRI	0.71±0.09	0.82±0.05	0.90±0.15	0.32±0.26
Exp V: Real CT + Real MRI	0.71±0.08	0.84±0.03	0.96±0.05	0.17±0.11

**Figure 5:**
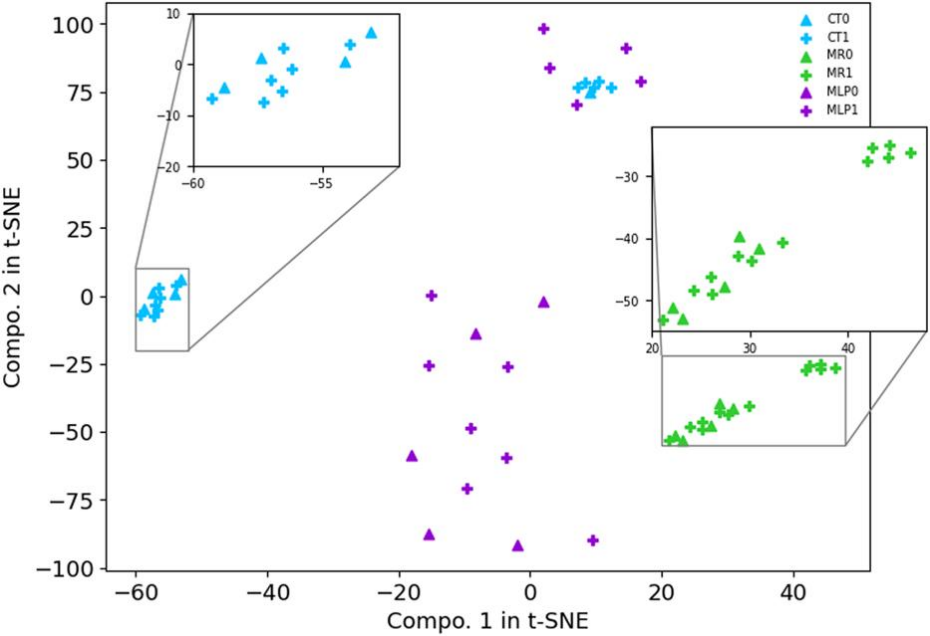
**t-SNE visualization of three embeddings with DS3 (mixed) test subjects (12 good recovery versus 5 bad recovery).** They are from the last layer of real CT (CT0 and CT1) and synthetic MRI (MR0 and MR1) ResNet-18 models and the last layer of MLP (MLP0 and MLP1). Here, 0 indicates bad recovery while 1 indicates good recovery. The *x*- and *y*-axes represent the first and second components from t-SNE, respectively. The blue triangle denotes CT embeddings with predicted bad recovery, while the blue cross indicates CT embeddings with predicted good recovery. Similarly, the green triangle represents MR embeddings with predicted bad recovery, and the green cross represents MR embeddings with predicted good recovery. The purple triangle corresponds to MLP embeddings (a combination of CT and MR) with predicted bad recovery, and the purple cross corresponds to MLP embeddings with predicted good recovery.

In Fig. 5, we present the visualization of embeddings from the last layer of the ResNet-18 models trained on real CT (shown in blue, corresponding to the blue rectangle in Fig. 2) and synthetic MRI (shown in green, corresponding to the green rectangle in Fig. 2). Additionally, we visualize the embeddings from the final layer of the MLP, which processes the concatenated embeddings of real CT and synthetic MRI (shown in purple, corresponding to the purple rectangle in Fig. 2). All embeddings are from Experiment IV. The visualization on the DS3 (mixed) test subjects (12 good recovery versus 5 bad recovery) is achieved by t-distributed stochastic neighbour embedding^37^ (t-SNE) to show this complementary effect. It is observed in the embedding space (i) real CT presents two separatable clusters: one cluster has 7 good recoveries versus 4 bad recoveries, and one has 5 good recoveries versus 1 bad recovery; (ii) synthetic MRI also presents two clusters though less distinguishable: one cluster has 7 good recoveries versus 5 bad recoveries, and one cluster has 5 good recoveries; (3) MLP (joint real CT and synthetic MRI) shows the trade-off between the real CT and synthetic MRI embedding showing the two clusters with one cluster having 7 good recoveries and 5 bad recoveries, the other has 5 good recoveries. We contend that although the embeddings of real CT and synthetic MRI differ significantly, both can be leveraged to improve the separability between good and bad recovery samples in the MLP model. This demonstrates the complementary effect between CT and synthetic MRI for outcome prediction. Given the small size of the test set, even a few misclassifications can lead to noticeable fluctuations in performance metrics such as AUC. While it may seem surprising that Exp IV slightly outperforms Exp V, we interpret this as evidence that the synthetic MRI captures key structural features that are relevant for outcome prediction. Rather than claiming superiority, our goal is to demonstrate that synthetic MRI can approximate the utility of real MRI in this context.

The model trained with a combination of real MRI and real CT achieves a higher *F*1-score, which is less affected by class imbalance, compared to the model trained using either real MRI or real CT alone. This supports our hypothesis that CT and MRI provide complementary information. Since there are only five negative (bad recovery) samples in the testing set, specificity estimates are inherently susceptible to high variance, where a single misclassification can lead to noticeable fluctuations. Consequently, small decreases in specificity may be less reliable as performance indicators. However, the significant improvement in sensitivity and overall performance metrics, such as *F*1-score and AUC, suggests that our method provides a more robust and effective prognostic approach. While specificity exhibits minor variability, the overall trend indicates that integrating synthetic MRI with CT enhances model robustness in real-world applications.

## Discussion

The main finding of this study is that the prediction of patient outcomes 3 months following TBI is facilitated by using a DL-based harmonization model that integrates synthetic brain MRI with real brain CT. This method could improve the ability and accuracy to predict patient outcomes in the clinical setting since brain CT is more commonly available than brain MRI. Predicting patient outcomes following TBI can help determine which patients should receive early intervention, the frequency and intensity of follow-up evaluations and the need to refer patients to clinicians with special expertise in TBI. Furthermore, early prediction of TBI outcomes could assist with determining patient eligibility and/or stratifying patients for clinical trials investigating TBI treatments.

Numerous prior publications have reported the value of brain imaging for predicting patient outcomes after TBI. For example, head perfusion CT and structural CT findings associate with patient outcomes up to 12 months after an mTBI^38-41^ and following severe TBI. Outcome prediction using data from the Medical Research Council CRASH (Corticoid Randomization after Significant Head Injury) Trial demonstrated that head CT findings, such as petechial haemorrhages, intracranial haemorrhage, midline shift and obliteration of the third ventricle or basal cisterns, contribute to accurate prediction.^42^ Pease *et al.*^9^ utilized CT scans in combination with various clinical inputs such as race, sex and mechanism of injury to predict outcomes in patients with severe TBI, employing a multi-modality learning approach that integrates imaging and clinical tabular data. Other studies have demonstrated the value of brain MRI for classifying and prognosticating outcomes amongst those with TBI, including structural and functional sequences.^7,11,43^ Several reviews that summarize the use of brain imaging for TBI prognostication have been published.^44-46^ Our pilot study adds to the existing literature by integrating synthetic brain MRI for participants who otherwise only have brain CT, taking advantage of the complementary information provided by each imaging modality without actually requiring completion of the brain MRI, which can be costly.

In the prediction model, effectively handling the two embeddings generated by the ResNet models is critical. We maintained the original embedding size of 1024 after concatenation. This combined embedding is then processed by an MLP consisting of two fully connected layers, each with a width of 1024, which yielded the best performance. We also experimented with an alternative fusion approach, such as concatenating the two modalities at the input stage to create a two-channel input for a single ResNet model. However, this approach led to one modality consistently dominating the other, reducing prognosis effectiveness. The width of the fully connected layer is also obtained by grid search.

There was limited availability of paired data for verifying the prognostic model’s performance. To mitigate this issue, we applied resampling on imaging samples associated with poor patient recovery during training and implemented a 5-fold CV strategy to ensure more robust results. However, we acknowledge that a larger dataset would further validate the effectiveness of our approach and strengthen our findings. The TRACK-TBI Pilot dataset is a valuable and widely used resource that has significantly contributed to TBI research. However, as with many large-scale public datasets, there are some challenges related to image quality. For example, a small subset of images may be incomplete (e.g. only partially covering the brain) or contain imaging artefacts that can affect downstream analyses. To ensure optimal performance of harmonization models, we recommend implementing a thorough quality control process prior to training. In our study, we observed that the l1 distance and SSIM, used to assess harmonization quality, were lower than expected, likely influenced by unpaired training. Furthermore, the limited visual overlap is a known limitation of current state-of-the-art unpaired image-to-image translation methods. Because the model is trained without paired data, fine-grained structural details may not be fully preserved during translation.^47,48^ We suggest this as an area for future investigation. Another challenge in this pilot study is that the number of unpaired MRI data is significantly lower than that of unpaired CT in TRACK-TBI Pilot dataset. Due to the limited number of MRIs available for FP-GAN training, the model may have restricted feature space exploration, potentially leading to bias. We acknowledge this limitation and plan to address it in future work by leveraging generative models, e.g. diffusion models, which are widely used in medical applications,^49-52^ to augment the dataset.

We also performed classification exclusively on mTBI patients by removing other types of TBI. In this setting, a larger proportion of poor recovery samples were excluded, which may have introduced a risk of overfitting. We observed that experiments using either real or synthetic MRI achieved higher *F*1-scores and sensitivity, indicating better detection of good recovery cases compared to the experiment using CT alone. This suggests that CT may be less effective for outcome prediction in mTBI and MRI is a better choice. Notably, the mTBI and mixed TBI datasets contained only four and five poor recovery samples, respectively, meaning that even a single misclassification can lead to a substantial drop in specificity. Nonetheless, the specificity across experiments suggests that our model is not significantly overfitting because the model continues to predict both classes with non-trivial accuracy, as evidenced by high sensitivity, moderate-to-high *F*1-scores and improvements in AUC. Additionally, across different test settings and folds, we observe variance in predicted outcomes, indicating that the model has learned meaningful discriminative features rather than memorizing patterns from the training set.

## Conclusion

In this study, we employed the FP-GAN model for harmonizing CT and T1-MRI data. We utilized CT scans along with their corresponding synthesized T1-MRI for predicting patient outcomes at 3 months following TBI. The prediction results demonstrate that incorporating synthetic MRI significantly enhances performance compared to using CT alone.

## Data Availability

All data used in this study were obtained from the Federal Interagency Traumatic Brain Injury Research (FITBIR) Informatics System. Access to the FITBIR datasets requires proper authorization and adherence to their data use agreements. Researchers interested in accessing the data can apply through the FITBIR Data Access Request process. Data sharing is not applicable to this article as no new data were created or analysed in this study. Model code, training scripts and pre/postprocessing pipelines are available at: https://github.com/SoloChe/TBI-Recovery-Prediction-Harmonization.
